# New Method of Reduction of Dislocation of the Hip

**Published:** 1877-11-20

**Authors:** S. J. Allen

**Affiliations:** White River Junction, Vt.


### NEW METHOD OF REDUCTION OF DISLOCATION OF THE HIP.

By S. J. ALLEN, M. D., White River Junction, Vt.

One day in the month of March, 1841, at which time I was a student of medicine in the office of John L. Swett, M. D., of Newport, N. H., I was riding in my sleigh, about three miles south of the village, and, passing a house situated some six rods from the road, I heard an outcry, Looking in the direction of the alarm, I saw a woman, Mrs. Perry by name, who, in stepping from the door, had slipped and fallen upon the icy ground. Hitching my horse, I walked rapidly towards her. As I came near, two men came out of the house, and, lifting her erect, assisted her inside. While they were bearing her along, I noticed that the right foot turned in upon the dorsum of the left; and I said to myself—"Case of dislocated femur upon the dorsum illii." Expressing my opinon to the friends of the woman, I said, "You must send for Dr. Swett to reduce it." A messenger was directly dispatched, who upon the way met Dr. Mason Hatch, a respectable practitioner of medicine, but less skilled in surgery. The doctor being requested to call, did so. and examined the hip by passing his hand over it, saying that he guessed the hip was not out of joint; and bringing from his sleigh a box of Kittridge's ointment, directed it to be applied three times a day to the hip, saying at the same time that he thought the patient would be well in a few days. After the learned doctor's departure, I repeated my opinon that the hip was dislocated, and that Dr Swett must be summoned to put matters right. While the horse was being harnessed the second time, I concluded to make some examination of the limb for the purpose of reassuring myself of the correctness of the diagnosis. Grasping the leg with my right hand, I flexed the leg upon the thigh, and the thigh at right angles with the body. The old lady, for thus I considered her then, although but forty, complained of my hurting her; and some how the limb became fixed in the position, and could not well be moved. It seemed locked, and could not be moved further without considerable force and pain. With the view of relieving my patient from this uncomfortable state, I stepped upon the bed, standing with her limb between my own limbs, and placing the dorsum of her foot upon my nates, and my right hand under the bend of her knee, I lifted her hips from the bed, holding her steadily in that position a few seconds, when the head of the dislocated bone slipped into the socket, accompanied by that peculiar audible shock which so delights the surgeon's ear. She immediately exclaimed, "I am well! I am well!" Of course, it was unnecessary to send for Dr. Swett now, so the horse was returned to the stable.

On my return to Newport village, I found Dr. Kittridge, of Claremont, N. H., present, with Dr. Swett, and I immediately related the incident as above described. I was informed by the two justly eminent surgeons that it was not a case of complete luxation, but that the head of the femur got caught upon the edge of the acetabulum, and that my manipulation had, fortunately and accidentally lifted the bone into the socket. This announcement made my hat and coat seem very small; but I accepted the situation with a submissive grace, although I never forgot the method of reduction. Keeping it in mind, I intended to apply it in my next case, which I confidently expected to meet with sooner or later.

September 21, 1848, I was called to a little girl ten years old—Minnie Clark—who, while climbing upon a heavy gate resting upon the fence, fell upon her back, the gate falling upon her, dislocating the right femur upon the dorsum illii. I attempted reduction by the method resorted to in the case of Mrs. Perry, but failed, in consequence of the great rigidity of the muscles, the light weight of the body of the child, and the want of an anesthetic. So I sent for Dr. Dixi Crosby, who reduced it with Jarvis's adjuster, after saying that he disliked to apply so powerful an instrument to so young a subject, fearing that he might separate the epiphysis at some point. Jarvis' adjuster was very generally used at that time, and in that vicinity, to reduce dislocations.

The 16th of July, 1872, I was called, in consultation with Dr. Sperry, or West Hartford, Vermont, in the case of a French Canadian, Lewis Baumhar, a section hand on the Central Vermont Railroad, who, while helping to carry a track-rail, fell on his right knee, the rail slipping from his shoulder and falling upon the sacrum, dislocating the right femur upon the dorsum illii. When I arrived, Dr. Sperry asked me if I had my pulleys with me. I answered that I had the pulleys which the Almighty furnished me with. Said the doctor, "You can't set the leg without pulleys." I answered that I could try. After the patient was fully chloroformed, the muscles being thorougly relaxed, I stepped upon the bed, and flexed the leg upon the thigh, and the thigh at right angles with the body, and placing his foot between my legs, and my hand beneath the bend of his knee, I lifted the hips well from the bed, and held them immovable in that position less than half a minute, when the head of the thigh bone returned into the socket with a sensible and audible shock. The reduction was accomplished so quietly that the doctor did not notice when it occurred, nor did he understand the method used, and at first questioned the fact of its having been reduced.

September 25, 1874, I was called, with Dr. Davis, of Lebanon, New Hampshire, in the case of N. S. Huntington, of Hanover, New Hampshire, a brakeman on the Central Vermont Railroad, who, while coupling cars at Claremont Junction, had his right hip dislocated on the dorsum. Chloroform was administered by Dr. Davis, and I reduced this dislocated femur in the same manner as in the case of Baumhar, and with the same facility.

January 13, 1877, called, in consultation with Dr. B. F. Eaton, of Hartford, Vermont, to see A. Woodbury, a freight brakeman, who had his left hip dislocated while coupling cars at Bellows Falls, Vermont. Dr. Eaton gave the chloroform, while, by the same method as in the above described cases, I returned the dislocated bone to its proper place in less than half a minute.

These four cases are all I have to relate as testing this new, easy, and, I claim, unfailing method of reducing luxations of the hip joint. It will be noticed that they are all cases of dislocation on the dorsum illii; but, at the same time, we should be reminded that the dislocation on the dorsum is the type of all luxations of the femur, and that before the reduction is accomplished in other and rarer forms, the head of the thigh bone is thrown on the dorsum by manipulation before it can be returned to the acetabulum. Indeed, it is not uncommon for the head of the femur to be changed from one position to the other several times during the manipulations before it can be returned to the socket, in cases of pubic and ischiatic forms of displacement, by the method of Nathan Smith.

By my method, the lower part of the body is lifted from the floor and held immovable. This maneuver relaxes the femoral ligament, and the weight of the hips and opposite limb rotates the body outward, producing sufficient abduction and extension to quietly draw the acetabulum over the head of the femur, and at the same time compelling the patient to become "particeps criminis" in case of a suit for mal-practice.—*Ohio Med. and Surg. Journal.*

